# The Best Stoma in an Emergency

**DOI:** 10.1007/s00268-023-07151-w

**Published:** 2023-09-05

**Authors:** Su Mei Hoh, David Allan Watters

**Affiliations:** 1 School of Medicine and Health Sciences, Deakin University, Geelong, Waurn Ponds, Victoria Australia; 2 University Hospital Geelong, Barwon Health, Geelong, Australia

In emergency colorectal surgery, a primary anastomosis may be unwise or require protection, and so the formation of a stoma can be a lifesaving maneuver. Despite the proven value of preoperative stoma site planning, urgency and patient condition often result in the operation proceeding without it occurring. However, a poorly sited stoma can result in a life of misery from ongoing stomal complications.

In this issue of the journal, Watanabe et al.’s paper looks at the impact of preoperative stoma marking on early outcomes following emergency colorectal surgery [1]. This is an important topic given 20–50% of stomas formed at an emergency operation never get reversed, particularly when formed as part of a Hartmann’s procedure. Their large retrospective cohort study was based on administrative data largely obtained from academic hospitals and grouped complications as stoma specific, surgical and medical. They found that preoperative stoma siting did not impact overall early morbidity and mortality, though they did not have access to longer term outcomes after hospital discharge. Their 4.7% 30-day mortality rates are impressive.

There has been much work on the risk factors associated with stomal specific complications including stomal marking. A meta-analysis of over 10,000 patients reported a pooled incidence of stomal complications in 35%, with obesity, laparoscopic stoma formation and emergency surgery being the most prominent risk factors [2]. Other patient-related risk factors include male gender, age > 60 years, ASA >3 and the presence of chronic disease such as diabetes [2]. There is also some indication that alcohol consumption and smoking contribute to stomal complications. At emergency surgery, almost all of these risk factors are not modifiable, and the urgency of the surgery itself is a major risk factor for morbidity. While the rate of preoperative stomal siting is low, particularly in the emergency setting [1], this is one modifiable risk factor for stoma morbidity.

There are a few specific challenges to preoperative stomal siting prior to urgent surgery. The first being time of day and the availability of a stomal therapist to mark the patient. In the absence of a stomal therapist, few surgeons or surgical trainees have been formally trained to site a stoma [3]. There is also no consensus as to the optimal method of stomal siting. A more common method is placing the stoma along a line drawn from the umbilicus to the anterior superior iliac spine (ASIS) either halfway or one third of the way, preferably through the rectus sheath [3]. Ideally, a patient is assessed in multiple positions (sitting, standing, bending) to ensure that the stoma site is visible and away from any skin creases and bony prominences [3]. As Watanabe points out, this can be difficult to achieve as the emergency surgeon is often faced with a peritonitic patient who can only be marked lying down or already draped on the operating table.

Siting a stoma away from skin creases is important to allow for proper attachment and sealing of pouching systems. A recent meta-analysis by Hsu et al. found that preoperative siting was related to a reduced incidence of skin complications, such as dermatitis and ulceration, with some series showing a twofold decease in complications [4]. Visibility of the stoma is important if patients are to achieve independent stomal care. Achieving visibility away from creases is a particular challenge in the patient with higher abdominal adiposity and girth. Placement of a stoma in the usual lower quadrants where there is a large abdominal pannus can be problematic. The pannus itself could obstruct direct view. Also, when upright, the gravitational fall of the pannus tends to pull the stoma downward away from view. Optimal placements of stomas for the morbidly obese need to have trephines made higher and often encroaching into the upper quadrants. Skin complications were not identified at a higher rate in Watanabe’s series. However, these ongoing self-care issues are often managed in the community setting by the stomal therapy nurses which, as the authors acknowledge, are not captured in this series that focuses on inpatient occurrences [1].

The most dreaded late peristomal complication is the parastomal hernia, which occurs in a third of patients with a stoma, regardless of surgical urgency or indication. They develop months or years after the stoma-forming surgery. Their incidence was not captured in Watanabe’s series, which was based on the in-patient admission. Hsu et al.’s [4] meta-analysis found that preoperative marking does reduce the incidence of parastomal hernias. Stoma aperture size and patient age are independent risk factors for parastomal hernias [5]. Every extra millimeter of aperture size increases the stomal hernia incidence by 10% [5]. In the emergency setting, it is not uncommon to require a larger fascial trephine to facilitate the exteriorization of colon particularly when there is distention and congestion from an obstruction. Larger apertures must then be tolerated to avoid injury or strangulation of edematous bowel before congestion subsides postoperatively.

A poorly formed stoma affects quality of life and can have detrimental long-term social and psychological impacts. Complications can also increase the health care costs resulting in further surgery, hospital readmissions, increased length of stay, increased consumption of medical supplies and need for community nursing [2]. Further studies on the effect of stomal marking in the emergency patient with longer follow-up into the community setting will be useful.

Although one cannot preoperatively perform stoma site marking for all emergency colorectal surgery patients, the value of this should not be forgotten and every effort made to do it when possible. Surgical education and training should include more formal assessment of stoma site marking, particularly in the emergency setting [3]. Stoma formation should not be delegated to an unsupervised, junior member of the surgical team. At least there should be direct supervision of the placement and formation of the stoma by the operating senior surgeon as this will reduce early and late complications of a stoma and result in a better quality of life for surviving patients even if they choose not to undergo a reversal.
